# Increased expression of serine palmitoyl transferase and *ORMDL3* polymorphism are associated with eosinophilic inflammation and airflow limitation in aspirin-exacerbated respiratory disease

**DOI:** 10.1371/journal.pone.0240334

**Published:** 2020-10-08

**Authors:** Ga-Young Ban, Dong-Ye Youn, Young-Min Ye, Hae-Sim Park

**Affiliations:** 1 Department of Pulmonary, Allergy and Critical Care Medicine, Kangdong Sacred Heart Hospital, Hallym University College of Medicine, Seoul, Korea; 2 Allergy and Clinical Immunology Research Center, Hallym University College of Medicine, Seoul, Korea; 3 Department of Allergy and Clinical Immunology, Ajou University School of Medicine, Suwon, Korea; Flinders University of South Australia, AUSTRALIA

## Abstract

**Background:**

Patients with aspirin-exacerbated respiratory disease (AERD) are known to have poor clinical outcomes. The pathogenic mechanisms have not yet been completely understood.

**Objective:**

We aimed to assess the involvement of the *de-novo* synthetic pathway of sphingolipid metabolism in patients with AERD compared to those with aspirin tolerant asthma (ATA).

**Methods:**

A total of 63 patients with AERD and 79 patients with ATA were enrolled in this study. Analysis of mRNA expression of serine palmitoyl transferase, long-chain base subunit 2 (SPTLC2) and genotyping of *ORMDL3* SNP (rs7216389) was performed.

**Results:**

Significantly higher levels of SPTLC2 mRNA expression were noted in patients with AERD, which showed significant positive correlations with peripheral/sputum eosinophil counts and urine LTE_4_ (all *P*<0.05). The levels of SPTLC2 mRNA expression showed significant negative correlations with the level of FEV_1_ and FEV_1_/FVC (*P* = 0.033, *r* = −0.274; *P* = 0.019, *r* = −0.299, respectively). Genotype frequencies of *ORMDL3* SNP (rs7216389) showed no significant differences between the AERD and ATA groups. Patients with AERD carrying the TT genotype of *ORMDL3* had significantly lower levels of FVC (%) and PC_20_ methacholine than those carrying the CT or CC genotype (*P* = 0.026 and *P* = 0.030).

**Conclusion & clinical relevance:**

This is the first study that shows the dysregulated *de novo* synthetic pathway of sphingolipids may be involved in the eosinophilic inflammation and airflow limitation in AERD.

## Introduction

Asthma is a heterogeneous disease characterized by chronic airway inflammation affecting 1%-18% of the general population worldwide [1]. Asthmatics present variable clinical symptoms of wheeze, shortness of breath, cough, chest tightness, and airflow limitation. Airway inflammation is the most important feature of asthma which involves several inflammatory cells including eosinophils, lymphocytes, mast cells, and neutrophils [2]. Airway inflammation exacerbates airway obstruction by promoting mucus secretions, mucosal edema, and airway hyperresponsiveness which finally cause airway remodeling.

Aspirin-exacerbated respiratory disease (AERD) is characterized by persistent asthma and chronic rhinosinusitis with nasal polyp as well as aspirin/nonsteroidal anti-inflammatory drug (NSAID) hypersensitivity, where eosinophilia is a common finding in the upper and lower airway mucosa [3]. A recent meta-analysis reported that the prevalence of AERD is 7% in adult asthmatic patients and 14% in patients with severe asthma [4]. The risk of uncontrolled asthma, severe asthma and asthma exacerbation is reported to be higher in patients with AERD than in those with aspirin tolerant asthma (ATA) [5]. Identifying patients with AERD is important for education of aspirin/NSAID avoidance and leukotriene modifier treatment because of its high morbidity.

Traditionally, dysregulation of arachidonic acid metabolism is well-known pathophysiology involved in AERD. Baseline levels of leukotriene E_4_ (LTE_4_) have been reported to be higher in patients with AERD than in those with ATA, which increased after aspirin challenge [6]. A recent meta-analysis demonstrated that urinary LTE_4_ can be used as a potential biomarker for identifying AERD [7]. Cysteinyl leukotrienes (CysLTs) can cause eosinophil recruitment, mucosal edema, mucus secretion and bronchial constriction. In addition, the novel role of CysLTs has been proposed to trigger group 2 innate lymphoid cells, resulting in increased production of type 2 and other pro-inflammatory cytokines [8–10], whereas CysLTs directly boost group 2 innate lymphoid cell expansion through interleukin (IL)-33 and CysLT receptors, enhancing type 2/eosinophilic inflammation [9,10].

Alterations in sphingolipid metabolism have been reported to play a role in asthma pathogenesis [11–17]. Most studies have focused on the action of the metabolites in the recycling pathway of sphingolipid synthesis, especially, sphingosine-1-phosphate (S1P) which participate in the development of airway hyperreactivity, bronchoconstriction and airway remodeling [17–19]. Recently, we have also reported the increased levels of S1P and sphingosine in patients with AERD compared to those with ATA [16]. Regarding the *de novo* synthetic pathway of sphingolipids, genome-wide association studies revealed that single nucleotide polymorphism (SNP) of oroscomucoid-like protein 3 (*ORMDL3)* at the 17q21 locus increased the risk of asthma [20–22]. The TT genotype of *ORMDL3* (rs7216389) has been reported to be associated with asthma exacerbation and the severity of asthma [23,24]. A recent review addressed that *ORMDL3* has inhibitory effects on serine palmitoyl transferase (SPT) in physiological conditions, while it enhances ceramide formation and affects airway inflammation and hyperresponsiveness in pathological conditions [12,14]. However, most of the sphingolipid metabolite studies deal with allergic asthma, early-onset asthma and uncontrolled asthma. There have been few studies dealing with the *de novo* pathway of sphingolipids in patients with AERD. In the present study, we aimed to assess the involvement of the *de-novo* synthetic pathway of sphingolipid metabolism in patients with AERD compared to those with ATA.

## Methods

### Study subjects and sample collection

A total number of 63 patients with AERD and 79 patients with ATA were recruited for this study at Ajou University Hospital (Suwon, South Korea) and Kangdong Sacred Heart Hospital (Seoul, South Korea) from March 2018 to March 2019.

Asthmatics who have been diagnosed by allergy specialists according to the Global Initiative for Asthma guideline (GINA) 2020 were enrolled. Exclusion criteria were as follows: 1) asthmatics who are not maintaining asthma medications regularly and 2) asthmatics who experienced severe asthma exacerbation during the previous month. AERD was defined as a typical clinical history (recurrent exacerbations of upper or lower respiratory reactions after ingestion of aspirin/NSAIDs) and/or a positive response to the lysine-aspirin bronchial provocation test (Lys-ASA BPT). The Lys-ASA BPT was conducted with increasing doses of Lys-ASA solution up to 300 mg/mL using the method previously reported [25]. The asthmatics were in a stable condition and the levels of FEV_1_ were higher than 70% of the predicted value in the Lys-ASA BPT. All anti-asthmatic drugs, including leukotriene modifiers, were stopped for at least 3 days before the Lys-ASA BPT. The result of the Lys-ASA BPT was considered positive if FEV_1_% was decreased by more than 20% after the challenge. Subjects who showed negative results to the Lys-ASA BPT or denied any changes in upper or lower respiratory tract symptoms after ingestion of aspirin/NSAIDs were defined as ATA. A diagnosis of severe asthma was made according to the definition of international ERS/ATS guidelines [26].

Peripheral blood and urine samples were collected from the subjects in the morning when their asthma was in a stable state. Written informed consent forms were obtained from all the study subjects, and the study was approved by the Institutional Review Boards of Ajou University Hospital and Kangdong Sacred Heart Hospital (AJIRB-GEN-SMP-13-108 and KANDONG 2018-03-010-004, respectively).

### RNA isolation and quantitative real-time PCR

Total RNA was isolated from the whole blood by TRIzol reagents (Invitrogen, Carlsbad, CA, USA), and cDNA was synthesized from 1 μg of total RNA according to the manufacturer’s instructions using a ReverTra Ace qPCR RT Kit (ToYoBo, Osaka. Japan). Real-time PCR was performed using PowerUp SYBR Green Master Mix (Applied Biosystems, Carlsbad, CA, USA) on the ABI 7500 system (Applied Biosystems). Primers were designed at Primer 3 (version 4.1.0). The primer sequences were SPT, long chain base subunit 2 (SPTLC2): 5’-CCA GAC TGT CAG GAG CAA CCA T-3’(forward), 5’- TTC GTG TCC GAG GCT GAC CAT A-3’(reverse), glyceraldehyde 3-phosphate dehydrogenase (GAPDH), 5’- GTC TCC TCT GAC TTC AAC AGC G-3’(forward), 5’- ACC ACC CTG TG CTG TAG CCA A-3’(reverse). The relative values for target mRNA expression were calculated after normalization to the Ct value from GAPDH gene expression using the ddCt method.

### SNP identification and genotyping

Genomic DNA was isolated from the peripheral blood using the Puregene DNA purification kit (Gentra, Minneapolis, MN, USA), according to the manufacturer’s instructions. Genotyping of *ORMDL3* SNP (rs7216389) was performed as previously described, according to the primer extension method using a SNaP shot ddNTP primer extension kit (Applied Biosystems) [27].

### LTE_4_ analysis

The concentrations of LTE4 in the urine samples were analyzed using liquid chromatography-tandem mass spectrometry after solid phase extraction with Oasis HLB (Waters, Milford, MA, USA). LTE_4_-d_5_ was used as the internal standard for LTE_4_. Chromatographic separation was performed using a Waters Acquity UPLC system (Waters) with a Hypersil GOLD column (2.1 x 100 mm, 1.9 μm: ThermoFisher Scientific, San Jose, CA, USA) under gradient conditions. The mobile phases consisted of water in 0.1% formic acid (solvent A) and acetonitrile in 0.1% formic acid (solvent B) at a flow rate of 0.4 mL/min. Data were obtained using an API5500 triple quadrupole mass spectrometer (AB Sciex, Framingham, MA, USA) equipped with an ESI source. Negative electrospray ionization in the multiple reaction monitoring (MRM) mode was employed. The MRM was based on m/z transition of 438>333 for LTE_4_ and 443>338 for LTE_4_-d_5_. The calibration curve was linear over the range of 0.064 to 8 ng/mL with a coefficient of correlation (*r*) greater than 0.99 for all instances. The between-run precision and accuracy of the quality control samples (0.064, 0.192, 1.2 and 6 ng/mL) were less than 4.7% and 92.5%, respectively. For the quantitative determination of creatinine in urine samples, 10 μL of the urine sample were applied to Creatinine Parameter Assay Kit (R&D Systems, Minneapolis, MN, USA).

### Statistical analysis

Continuous variables were compared using Student’s *t* test, and Pearson’s chi-squared or Fisher’s exact test was used for categorical variables. *P* values for genotype frequencies were obtained by a generalized linear model adjusted for age and sex as covariates using the co-dominant, dominant and recessive models. Pearson’s and Spearman correlation analyses identified associations among continuous variables. These computations were performed using SPSS software, version 22.0 (IBM Corp., Armonk, NY, USA). GraphPad Prism 5.0 software (GraphPad Inc., San Diego, CA, USA) was used to produce graphs.

## Results

### Clinical characteristics of the study subjects

Table 1 shows the demographic and clinical characteristics of the study subjects. The number of asthmatics who had chronic rhinosinusitis was higher in AERD patients (*P*<0.001). The baseline levels of FEV_1_/FVC (%) were significantly lower in patients with AERD (*P* = 0.004). There were no significant differences in other variables between patients with AERD and ATA. None of the study subjects were regularly taking systemic corticosteroids.

**Table 1:** Demographic and clinical characteristics of the study subjects.

	AERD (n = 63)	ATA (n = 79)	*P* value
Age (year)	45.56 ± 14.49 (16–78)	47.32 ± 14.35 (18–82)	0.471
Sex (Female)	44 (69.8%)	60 (75.9%)	0.414
Chronic rhinosiusitis	46 (73.02%)	28 (44.4%)	<0.001
Atopy	30 (47.6%)	47 (60.3%)	0.134
Total IgE (KU/L)	432.77 ± 750.93	402.82 ± 697.62	0.809
Sputum eosinophil (%)	35.77 ± 38.55	34.62 ± 33.75	0.873
Peripheral eosinophil count (/μL)	499.26 ± 407.75	456.81± 468.55	0.572
Baseline FEV_1_(% Pred)	82.95 ± 18.57	86.35 ± 17.78	0.271
Baseline FEV_1_/FVC (%)	76.99 ± 10.16	82.19 ± 8.74	0.004
PC _20_ of methacholine (mg/mL)	4.09 ± 6.72	5.03 ± 6.11	0.485
Severe asthma	22 (34.9%)	21 (26.6%)	0.283

Continuous data are presented as mean ± SD. Dichotomous data are presented as number (%). AERD, aspirin exacerbated respiratory disease; ATA, aspirin tolerant asthma.

### Assessment of the mRNA level of SPTLC2 in patients with AERD and ATA

**Fig 1:**
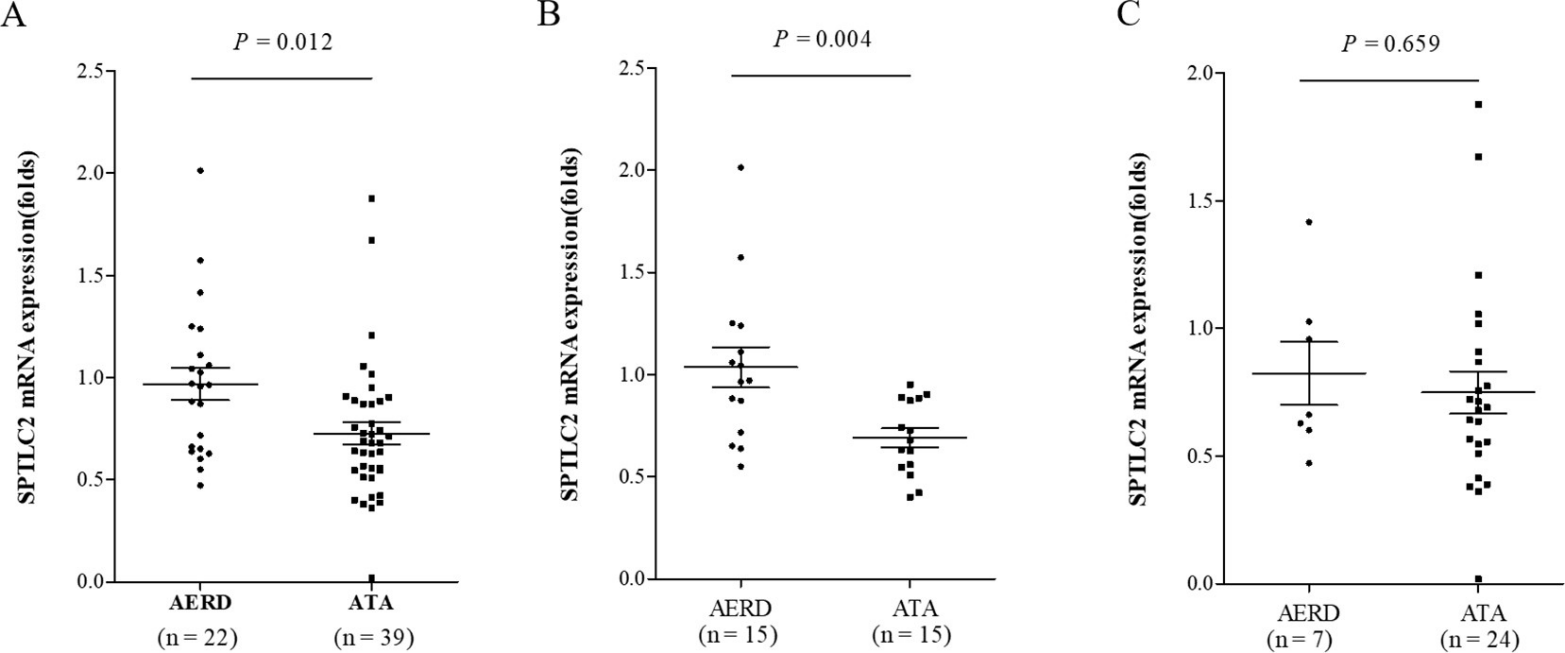
Comparison of the mRNA expression level of SPTLC2 between patients with AERD and ATA. (A) In study subjects with eosinophilic asthma. (B) In study subjects with non-eosinophilic asthma. AERD, aspirin exacerbated respiratory disease; ATA, aspirin tolerant asthma; SPTLC2, serine palmitoyl transferase, long-chain base subunit 2.

To assess the involvement of sphingolipids in the airway inflammation in AERD, the mRNA expression of SPT in blood was performed. SPT is known as the rate-limiting enzyme of the *de*-*novo* pathway of sphingolipid synthesis. The SPT complex is composed of the 3 different subunits SPTLC1, SPTLC2 and SPTLC3. SPTLC2 has generally been used to assess the SPT activity, especially with the SPTLC2 knockout model or the SPTLC2-specific inhibitor myriocin [14,19,21,28]. Therefore, this study focused on the evaluation of SPTLC2. The mean mRNA expression level of SPTLC2 in total asthmatics was 0.81 ± 0.37. It was significantly higher in AERD patients than in ATA patients (0.97 ± 0.37 vs. 0.73 ± 0.34, *P* = 0.012) (Fig 1A). AERD was a significant factor affecting the expression level of SPTLC2 even after adjustment for age and sex in linear regression analysis (*P* = 0.008, OR = 1.273). In the subgroup analysis of eosinophilic asthma (asthmatics with peripheral blood eosinophil count ≥ 300/μL), the mRNA expression level of SPTLC2 was significantly higher in patients with AERD than with ATA (1.04 ± 0.38 vs. 0.69 ± 0.18, *P* = 0.004). In non-eosinophilic asthma (asthmatics with peripheral blood eosinophil count < 300/μL), there was no significant difference between the 2 groups (Fig 1B and 1C).

### Correlations of clinical parameters with the mRNA expression of SPTLC2

**Fig 2:**
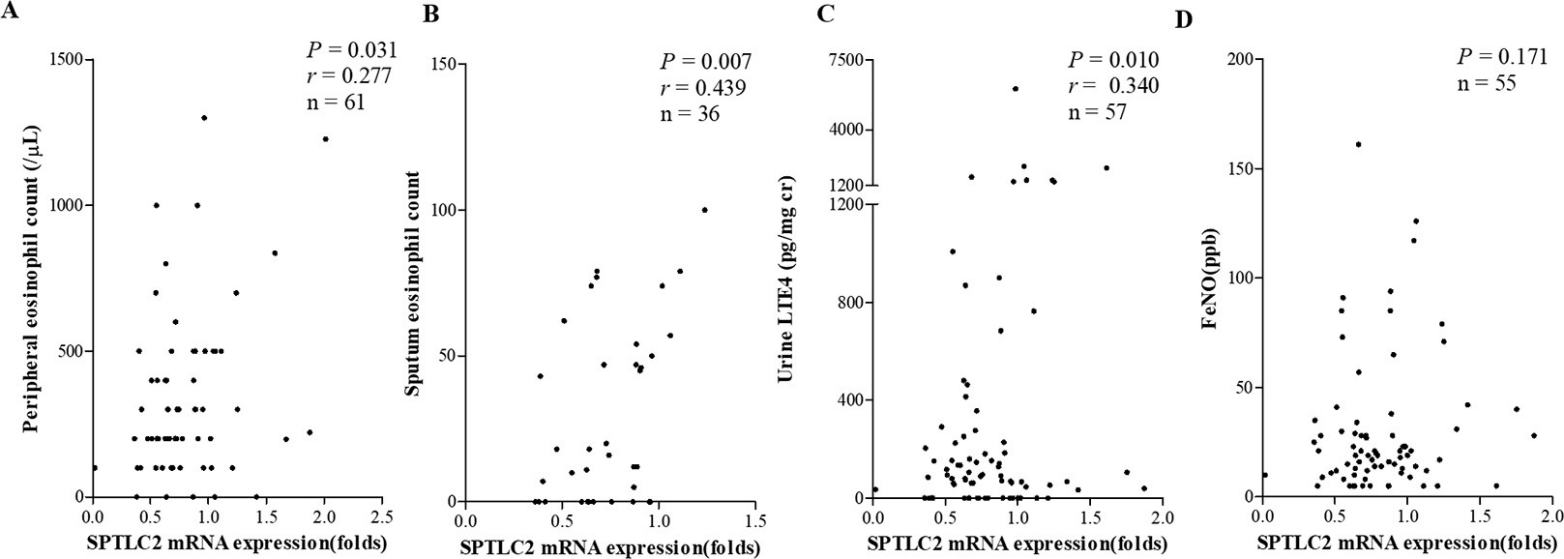
Correlations between mRNA expression levels of SPTLC2 and inflammatory parameters in the study subjects. Peripheral eosinophil count (A), sputum eosinophil count (B), urine LTE_4_ (C) and FeNO (D). LTE_4_, leukotriene E_4_; SPTLC2, serine palmitoyl transferase, long-chain base subunit 2.

**Fig 3:**
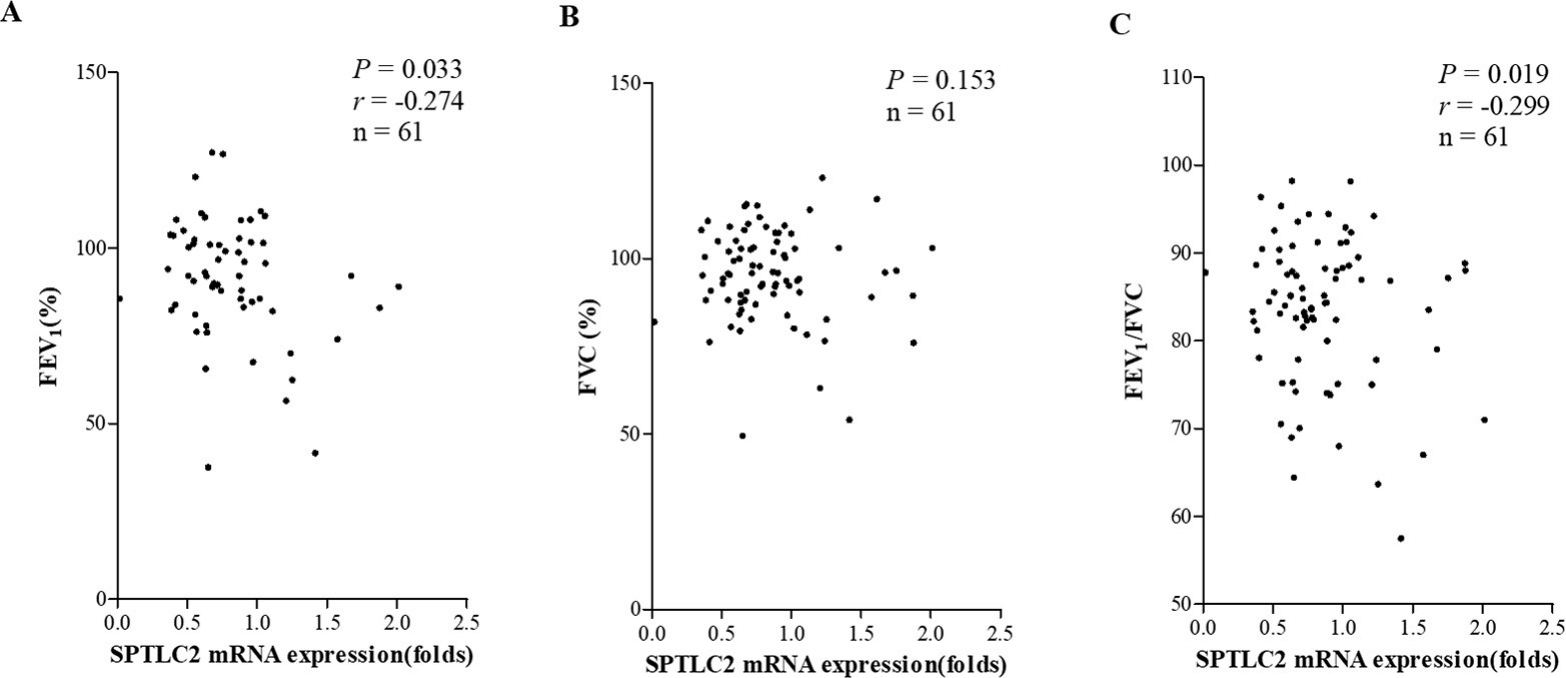
Correlations between mRNA expression levels of SPTLC2 and pulmonary function tests in the study subjects. Predicted level of FEV_1_ (%) (A), FVC (%) (B), FEV_1_/FVC (C). SPTLC2, serine palmitoyl transferase, long-chain base subunit 2.

**Fig 4:**
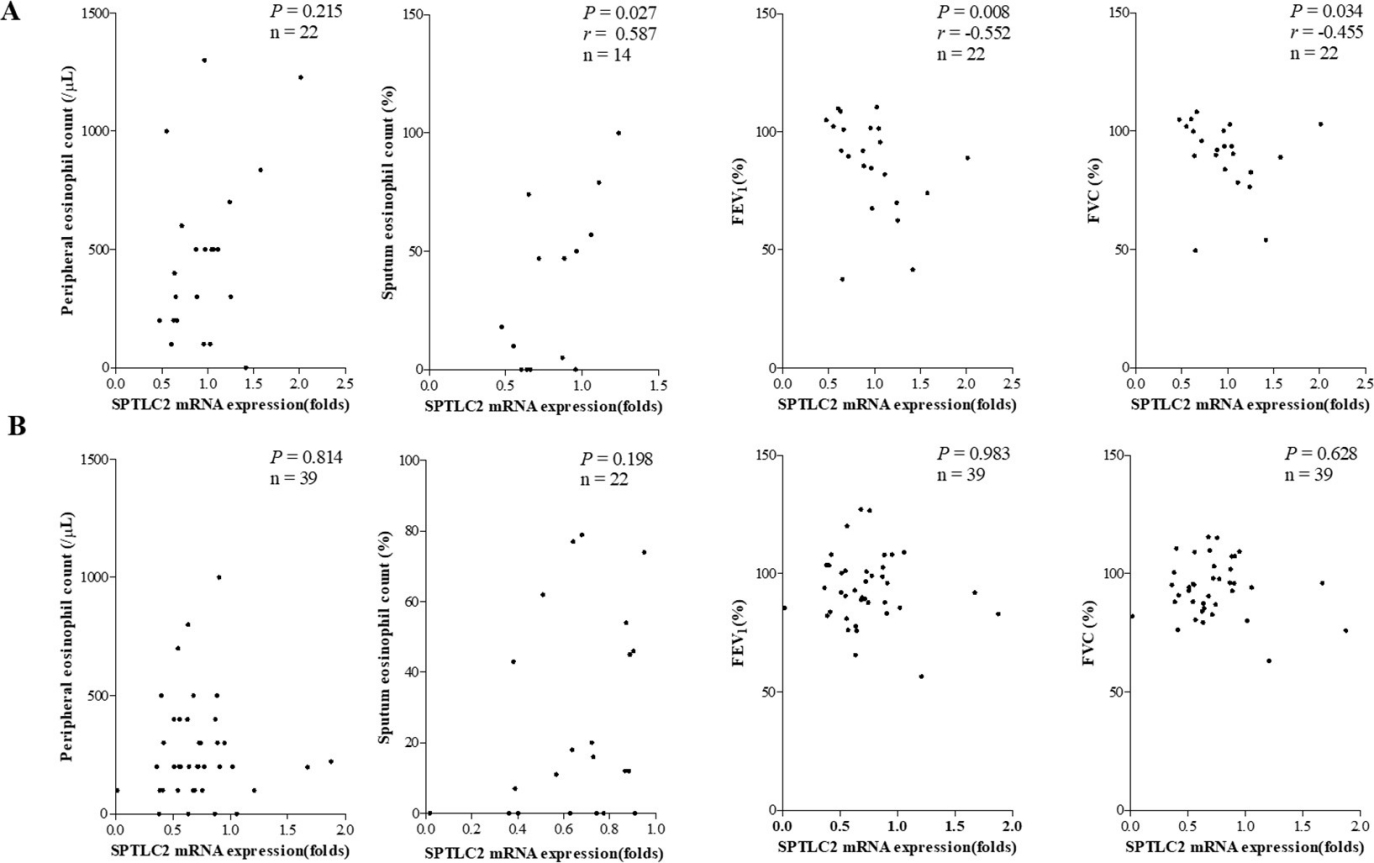
Correlations between mRNA expression levels of SPTLC2 and clinical parameters in patients with AERD (A) and those with ATA (B). AERD, aspirin exacerbated respiratory disease; ATA, aspirin tolerant asthma; SPTLC2, serine palmitoyl transferase, long-chain base subunit 2.

The mRNA expression levels of SPTLC2 showed significant positive correlations with peripheral blood eosinophil/sputum eosinophil counts and urine LTE_4_ (*P* = 0.031, *r* = 0.277; *P* = 0.007, *r* = 0.439; and *P* = 0.010, *r* = 0.340, respectively); however, they did not with FeNO (Fig 2). The predicted level of FEV_1_ (%) and FEV_1_/FVC (%) showed significant negative correlations between the mRNA expression levels of SPTLC2 in the study subjects (*P* = 0.033, *r* = −0.274 and *P* = 0.019, *r* = −0.299, respectively) (Fig 3). In patients with AERD, the mRNA expression levels of SPTLC2 positively correlated with sputum eosinophil counts and negatively correlated with the predicted level of FEV_1_ (%) and FEV_1_/FVC (%) (*P* = 0.027, *r* = 0.587; *P* = 0.009, *r* = −0.552; and *P* = 0.034, *r* = −0.455). Peripheral blood/sputum eosinophil counts, predicted level of FEV_1_% or FEV_1_/FVC (%) showed no correlation with the mRNA expression level of SPTLC2 in patients with ATA (Fig 4).

### Genotype frequencies for SNP within ORMDL3 (rs7216389) in patients with AERD and ATA

To evaluate the genetic effect of *ORMDL3* in susceptibility to AERD, the genetic polymorphism of rs7216389 in the *ORMDL3* gene was genotyped. Genotype frequencies were not significantly different between patients with AERD and ATA (Table 2). The rs7216389 did affect susceptibility to AERD, regardless of atopic status. In atopic asthmatics, the numbers of patients with AERD and ATA carrying TT, CT and CC genotype were as follows: 10,10 and 2 for AERD; and 9,6 and 3 for ATA, respectively (*P* = 0.427,0.406 and 0.511 in co-dominant, dominant and recessive analyses, respectively).

**Table 2:** Genotype frequencies for SNP within *ORMDL3* (rs7216389) involved in the sphingolipid metabolic pathway in patients with AERD and ATA.

Genotype	AERD	ATA	*P* value (OR)
	(n = 43)	(n = 42)	AERD vs. ATA
TT	24 (55.8%)	22 (53.7%)	0.226
CT	16 (37.2%)	13 (31.0%)	0.177
CC	3 (7.0%)	7 (16.7%)	0.860
T	64(74.4%)	57(67.9%)	0.398
C	22(25.6%)	27(32.1%)	

AERD, aspirin exacerbated respiratory disease; ATA, aspirin tolerant asthma.

### Clinical characteristics according to the genotype of *ORMDL3*

The levels of PC_20_ methacholine were significantly associated with the genotype of *ORMDL3* (rs7216389). Asthmatics carrying the TT genotype of *ORMDL3* showed significantly lower levels of PC_20_ methacholine than those carrying the CT or CC genotype (1.70 ± 2.01 vs. 5.10 ± 6.84, *P* = 0.043). The peripheral eosinophil count, FeNO, predicted value of FEV_1_, FVC or FEV_1_/FVC was not significantly different between asthmatics with the TT genotype and those with the CT or CC genotype.

**Fig 5:**
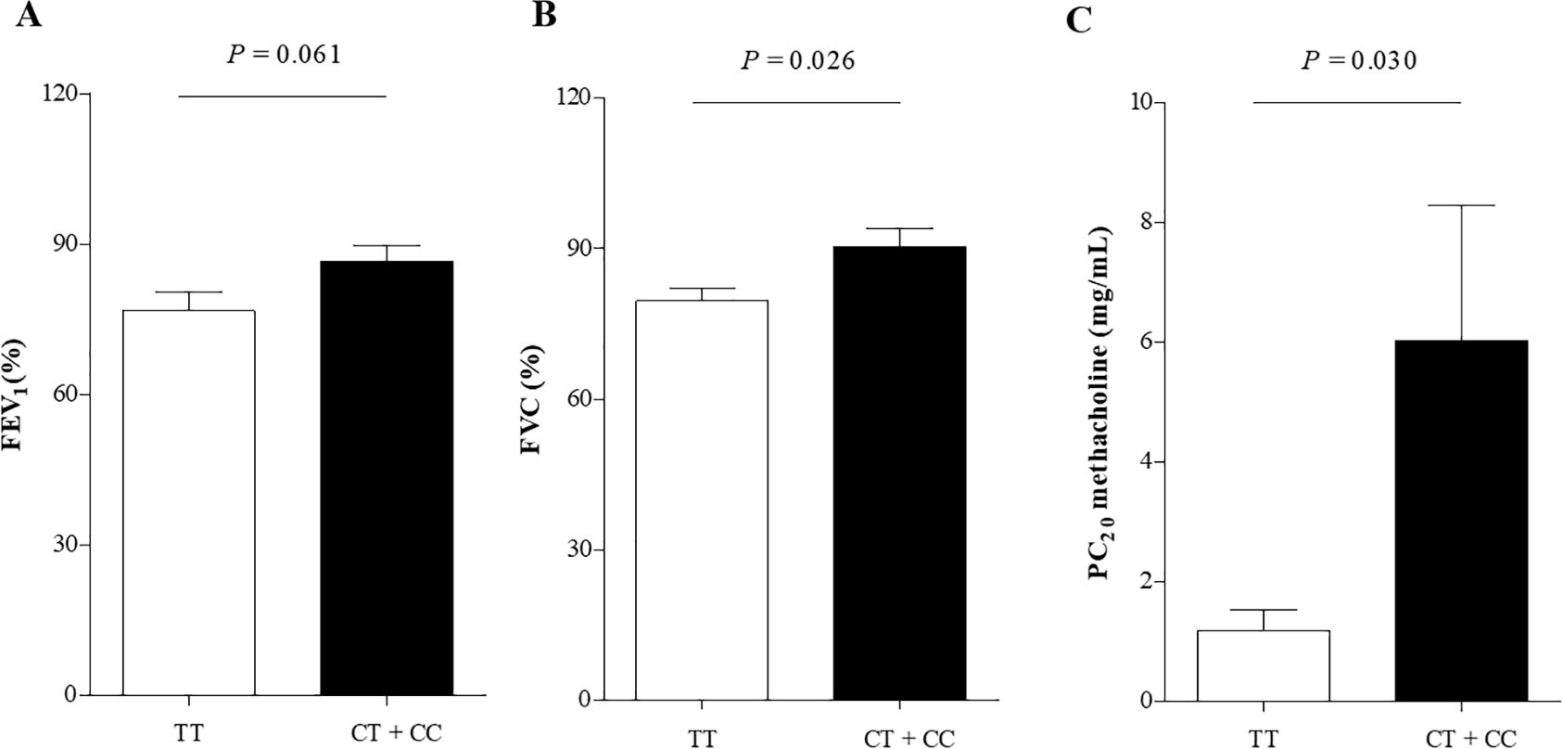
Comparison of lung functions according to the genotype of ORMDL3 (rs7216389) in patients of AERD. AERD, aspirin exacerbated respiratory disease; ATA, aspirin tolerant asthma.

To assess the genetic effect of *ORMDL3* on the clinical parameters of AERD, subgroup analysis was performed. Patients with AERD carrying the TT genotype of *ORMDL3* had significantly lower levels of FVC (%) and PC_20_ methacholine than those carrying the CT or CC genotype (*P* = 0.026 and *P* = 0.030, respectively) (Table 3 and Fig 5). The predicted level of FEV_1_ and MMEF tended to be lower in patients with AERD carrying the TT genotype, but did not reach the statistical significance. There were no significantly different parameters in patients with ATA according to the genotype of *ORMDL3*.

**Table 3:** Clinical characteristics according to the genotype of *ORMDL3* (rs7216389).

	AERD (n = 43)			ATA (n = 42)		
	TT (n = 24)	CT+CC (n = 19)	*P* value	TT (n = 22)	CT+CC (n = 20)	*P* value
Age (year)	42.46± 43.74	43.74 ± 12.58	0.772	47.05 ± 11.68	44.15 ± 14.23	0.474
Gender (Female)	15 (62.5%)	15 (78.9%)	0.244	17 (77.3%)	17 (85.0%)	0.524
Atopy	10 (41.7%)	12 (63.2%)	0.161	9 (40.9%)	9 (47.4%)	0.678
Total IgE (KU/L)	411.08 ± 502.43	700.94 ± 11887.10	0.330	500.14 ± 1063.42	425.63 ± 668.96	0.794
Peripheral eosinophil count (/μL)	605.28 ± 488.90	454.03 ± 367.75	0.269	484.55 ± 471.90	793.20 ± 620.70	0.079
FeNO (ppb)	47.75 ± 26.94	84.2 ± 62.01	0.269	43.20 ± 20.43	51.67 ± 46.05	0.707
Baseline FEV_1_(% Pred)	76.73 ± 18.24	86.51 ± 14.07	0.061	78.81 ± 16.43	80.18 ± 18.89	0.806
Baseline FVC (%)	79.51 ± 7.51	90.25 ± 9.06	0.026	81.45 ± 19.00	82.29 ± 14.29	0.921
Baseline MMEF (%)	35.79 ± 7.68	55.93 ± 21.35	0.069	62.45 ± 27.06	46.58 ± 18.97	0.188
Baseline FEV_1_/FVC (%)	73.27 ± 10.16	78.27 ± 8.78	0.130	81.68 ± 7.92	76.51 ± 9.44	0.218
PC _20_ of methacholine (mg/mL)	1.19 ± 1.46	6.03 ± 7.83	0.030	3.96 ± 2.81	3.73 ± 5.22	0.145

AERD, aspirin exacerbated respiratory disease; ATA, aspirin tolerant asthma.

## Discussion

This is the first report that observed the involvement of the *de*-*novo* synthetic pathway of sphingolipids in patients with AERD. We report here the increased expression of SPTLC2 mRNA (involved in the rate-limiting step in the *de novo* synthesis of sphingolipids) which closely correlated with the eosinophilic inflammation and airflow limitation in patients with AERD. Although the genotype frequencies for SNP within *ORMDL3* (rs7216389) showed no significant difference between AERD and ATA patients, AERD patients carrying the TT genotype of *ORMDL3* showed a lower level of lung function parameters and a higher degree of airway hyperresponsiveness than those carrying the CT or CC genotypes. Taken together, the *de*-*novo* synthetic pathway of sphingolipids may contribute to eosinophilic inflammation and airflow limitation in AERD.

Sphingolipids have been postulated to contribute tp asthma pathogenesis in terms of airway inflammation and bronchial hyperresponsiveness [29]. S1P was identified as a pathogenic contributor to asthma as well as a potent bioactive lipid molecule that regulates various cellular processes including cell growth, apoptosis and immune regulation [11,13,16]. These findings may represent pathophysiological changes during airway inflammation. Regarding the role of sphingolipids in the pathogenesis of AERD, we reported the increased serum levels of S1P and sphingosine in patients with AERD compared to those with ATA [16]. Ceramides in bronchoalveolar lavage fluid have been reported to be associated with airway inflammation and hyperreactivity in mouse models of house dust mite-induced asthma and neutrophil elastase [14,28]. A recent study demonstrated that the serum levels of ceramides were higher in asthmatic patients than in healthy controls and in asthmatic patients in an uncontrolled than in a controlled state [30]. Untargeted and targeted metabolomics study that aimed to determine different metabotypes according to asthma severity revealed that the serum levels of S1P, sphingosine and ceramides were high in severe asthmatics [31].

Despite accumulating evidence for the recycling synthetic pathway of sphingolipids [13,14,16,28,30,31], there are scanty data on the role of the *de novo* synthetic pathway of sphingolipids in asthma pathogenesis [29]. The pathogenic role of the *de novo* synthetic pathway has been suggested from the GWAS studies showing that *ORMDL* and *CerS2* were significantly associated with the risk of asthma [21,22,32,33]. In the *de novo* synthetic pathway of sphingolipids, SPT plays an important role along with ORMDL3 [29]. Pharmacological inhibition of SPT resulted in decreased levels of ceramides and eosinophil-related cytokines, such as IL-4, IL-13 and eotaxin, as well as eosinophil counts in bronchoalveolar lavage fluid [14,28]. The present study demonstrated that increased levels of SPTLC2 mRNA expression correlated with airway eosinophilia, urine LTE_4_ levels and airflow limitation in patients with AERD. Collectively, increased SPTLC2 may contribute to eosinophilic airway inflammation and lung dysfunction.

There have been numerous inconsistent and confusing mechanistic reports reporting on how *ORMDL* affects ceramide levels and airway pathophysiology [14,34–36]. A recent study suggested that moderate *ORMDL3* expression decreases ceramide levels in normal physiological conditions, while high *ORMDL3* expression increases ceramide levels, followed by increased inflammatory responses in pathological conditions [14]. In our genotype analysis, *ORMDL3* (rs7216389) showed no significant differences between the AERD and ATA groups. Meanwhile, consistent with the previous studies, the TT genotype of *ORMDL3* (rs7216389) is associated with asthma exacerbation and the severity of asthma [23,24]. The present study showed that AERD patients with the TT genotype had significantly lower levels of FVC (%) and PC_20_ methacholine than those with the CT or CC genotype, while ATA patients did not. Collectively, these findings imply that the genetic polymorphism of *ORMDL3* may affect gene expression and subsequently sphingolipid synthesis and can play a pathogenic role in airway hyperreactivity of AERD. Further studies are needed to evaluate the potential therapeutic effect in AERD management by inhibiting the *de novo* synthetic sphingolipid pathway.

Eosinophils are cardinal effector cells in asthmatic patients. Eosinophil infiltration in the sinus and bronchial mucosa has been recognized as major characteristics of AERD [3]. Patients with AERD had higher risk of uncontrolled asthma and suffered from severe asthma with more frequent asthma exacerbation than those with ATA [4,5]. Therefore, our finding of a close correlation between the *de novo* synthesis of sphingolipids and eosinophilic inflammation in AERD provides a new insight into the mechanism of AERD.

To our knowledge, this is the first study showing that the dysregulated *de novo* synthetic pathway of sphingolipids may be involved in the eosinophilic inflammation and airflow limitation in AERD. Although there have been several *in vitro* and mouse model studies regarding the *de novo* synthetic pathway of sphingolipid [14,37], we first reported direct evidence of close associations between the levels of mRNA expression, genetic polymorphism of relevant pathway and the degree of eosinophilic inflammation/lung function parameters in patients with AERD. However, due to the cross-sectional study design of our study, we cannot draw the conclusion of causal relationships between dysregulated sphingolipids and eosinophilic inflammation in AERD. The present study only focused on the initial stage of rate-limiting step in the *de novo* synthetic pathway of sphingolipids. Further studies to investigate the whole pathway of sphingolipid synthesis and to integrate the levels of metabolites, mRNA expression of individual enzymes, genotype frequencies and the degree of eosinophilic inflammation are warranted.

In conclusion, our results suggest that dysregulated *de-novo* sphingolipid metabolic pathways may play a crucial role in eosinophilic inflammation in AERD.

## Supporting information

S1 File 202005 Upload dataset. (XLSX)
